# DNase and atelectasis in non-cystic fibrosis pediatric patients

**DOI:** 10.1186/cc3544

**Published:** 2005-05-20

**Authors:** Tom Hendriks, Matthijs de Hoog, Maarten H Lequin, Annick S Devos, Peter JFM Merkus

**Affiliations:** 1 Pediatrician, Catharina Hospital, Eindhoven, The Netherlands; 2 Pediatric Intensivist, Division of Intensive Care, Department of Pediatrics, Erasmus University and Erasmus Medical Centre/Sophia Children's Hospital, Rotterdam, The Netherlands; 3 Pediatric Radiologist, Division of Radiology, Department of Pediatrics, Erasmus University and Erasmus Medical Centre/Sophia Children's Hospital, Rotterdam, The Netherlands; 4 Pediatric Pulmonologist, Division of Respiratory Medicine, Department of Pediatrics, Erasmus University and Erasmus Medical Centre/Sophia Children's Hospital, Rotterdam, The Netherlands

## Abstract

**Introduction:**

No evidence based treatment is available for atelectasis. We aimed to evaluate the clinical and radiologic changes in pediatric patients who received DNase for persistent atelectasis that could not be attributed to cardiovascular causes, and who were unresponsive to treatment with inhaled bronchodilators and physiotherapy.

**Methods:**

All non-cystic fibrosis pediatric patients who received nebulised or endotracheally instilled DNase for atelectasis between 1998 and 2002, with and without mechanical ventilation, were analysed in a retrospective descriptive study. The endpoints were the blood pCO_2_, the heart rate, the respiratory rate, the FiO_2 _and the chest X-ray scores before and after treatment.

**Results:**

In 25 of 30 patients (median [range] age, 1.6 [0.1–11] years) who met inclusion criteria, paired data of at least three endpoints were available. All clinical parameters improved significantly within 2 hours (*P *< 0.01), except for the heart rate (*P *= 0.06). Chest X-ray scores improved significantly within 24 hours after DNase treatment (*P *< 0.001). Individual improvement was observed in 17 patients and no clinical change was observed in five patients. Temporary deterioration (*n *= 3) was associated with increased airway obstruction and desaturations. No other complications were observed.

**Conclusion:**

After treatment with DNase for atelectasis of presumably infectious origin in non-cystic fibrosis pediatric patients, rapid clinical improvement was observed within 2 hours and radiologic improvement was documented within 24 hours in the large majority of children, and increased airway obstruction and ventilation–perfusion mismatch occurred in three children, possibly due to rapid mobilisation of mucus. DNase may be an effective treatment for infectious atelectasis in non-cystic fibrosis pediatric patients.

## Introduction

Atelectasis is a problem in many children with respiratory infections or who require ventilation. At least 8% of children on mechanical ventilation develop pulmonary atelectasis, with a concomitant increase in the morbidity and the length of stay [1]. There is no 'golden standard' for treatment of atelectasis in children. Efficacy of treatment modalities such as inhaled bronchodilators, steroids, physiotherapy and nebulised sodium chloride (NaCl 0.9%) has not been demonstrated [2].

Atelectasis is commonly caused by sputum blocking the airways. Mucus in patients with cystic fibrosis (CF) [3], in patients with bronchiectasis [4] and in patients with respiratory syncytial virus (RSV) bronchiolitis [5] contains significant amounts of extracellular DNA from degenerating leucocytes and epithelial debris. DNA increases the viscosity and adhesiveness of lung secretions [6]. Recombinant human DNase (rhDNase) has proven to be an effective treatment in opening airways in CF [7-9]. In infections complicated by atelectasis, bronchial secretions and mucus plugs also have a high concentration of DNA [5], such that DNase could also be an effective treatment in this situation.

Until now only case reports on the efficacy of DNase treatment in atelectasis have been published, suggesting efficacy [5,10-14] No randomised study has been published. The present study analysed the resolution of atelectasis following treatment with DNase in a large series of hospitalised children who were refractory to conventional treatment.

## Materials and methods

### Study subjects

This retrospective study included all patients who received rhDNase as treatment for atelectasis in patients with suspected or proven lower airway infection at Sophia Children's hospital, Rotterdam between 1998 and 2002. Patients were identified through the computerised pharmacy registration. Patients were included in the analysis when they had pulmonary atelectasis of at least one lobe, and when rhDNase was administered for that reason. CF patients were excluded and all other patients were evaluated. Thirty patients and 32 episodes of atelectasis were identified, and this included patients described previously [14]. When more than one atelectasis was treated, only the first episode was included in the analysis.

Table 1 presents the demographic data of the study group. The median age of the study group was 1.6 years. Sixteen patients were younger than 1 year of age; two of these patients were born prematurely at 26 weeks gestation, but were 6 months and 7 months corrected postnatal age at the time of admission. Sixteen patients were intubated in the days before receiving rhDNase (median, 3 days; range, 1–16 days). Twenty-five patients were treated in a pediatric intensive care unit, and five patients were treated in a medium care unit. Underlying illnesses or predisposing factors for severe lower airways infections were: airway malacia (seven patients), severe psychomotor retardation (five patients), congenital heart disease (five patients: three children with ventricular septal defect, one child with tetralogy of fallot, and one child with hypoplastic right ventricle, pulmonary atresia and blalock taussig shunt), tracheostomy (four patients), bronchopulmonary dysplasia (three patients), epilepsy (three patients), neuromuscular disease (two patients) and bronchiectasis (one patient). Because rhDNase was administered as a part of patient care and not as a medical trial, formal approval from an institutional review board or a medical ethics committee was not required in our hospital, and was therefore not requested.

### Methods

RhDNase was only considered and administered when patients did not demonstrate clinical improvement following empirical treatment for atelectasis and still demonstrated significant elevated work of breathing, could not be weaned easily off the ventilator or improved too slowly or not at all. RhDNase (Pulmozyme^®^; Roche, Basel, Switzerland) was administered either as a 2.5 mg dose nebulised twice daily with a jet nebuliser, using a tight-fitting mask and high-flow oxygen, in children breathing spontaneously, or 10% of this dose was diluted to 5 ml with NaCl 0.9% and given slowly as droplets during 30 min into the endotracheal tube or the canula twice daily. This treatment was continued until the atelectasis had improved sufficiently, preferably based on the chest X-ray (CXR) of the next day. This dose was chosen as it was estimated that pulmonary deposition of a regular 2.5 mg dosage would be a maximal 10%. When rhDNase was instilled endotracheally, it was attempted to position the head as favorably as possible for the DNase to reach the affected lobe(s).

RhDNase was administered twice daily until patients improved clinically because it was assumed that deposition in the peripheral airways of these children would be significantly diminished due to airway obstruction. All ventilated patients were sedated according to protocol, but were not paralysed, and the ventilators were standard not in the controlled ventilatory mode but in the pressure-regulated volume control mode. Blood gas control values were only obtained when the patients were stable, at least 30 min after manipulation or endotracheal suctioning.

Following rhDNase administration, the ventilator settings were not altered until the results of the blood gas analyses were available (2 hours later), except in the case of clinical deterioration. The nursing staff was only instructed to taper off FiO_2 _as much as possible. The clinical parameters analysed were the heart rate (HR), the respiratory rate (RR), the capillary or arterial pCO_2_, and the FiO_2 _before and within 2 hours following administration of rhDNase. Evidence for atelectasis on CXR was quantified using a CXR score before and within 24 hours of treatment with rhDNase. In children on mechanical ventilation, the peak inspiratory pressure, the mean airway pressure, days on the ventilator before receiving rhDNase and the time to extubation were recorded. All parameters and side effects were collected from patient files. In pediatric intensive care patients, dates were also obtained from the computerised data management system.

As cardiorespiratory endpoints we considered the change in clinical parameters (RR, HR, FiO_2_, PCO_2_) before and within 2 hours after the first dose of rhDNase, and considered the CXR score before and within 24 hours after the first dose of rhDNase. Parameters were compared using the Wilcoxon signed rank test. Interobserver comparisons of CXR scores were made using Cohen's kappa. Overall individual improvement in patients was defined as the improvement of two or more endpoints.

### Analysis

#### Clinical parameters

Parameters were compared before and within 2 hours after treatment with rhDNase.

Individual improvement of the FiO_2_, the RR and the HR was defined as >10% decrease, and deterioration was defined as >10% increase. Individual improvement of pCO_2 _was defined as a decrease >1 kPa, and deterioration was defined as an increase >1 kPa. When patients were on mechanical ventilation, the peak inspiratory pressure and the mean airway pressure improvement was defined as >3 cmH_2_O decrease of pressure and their deterioration was defined as >3 cmH_2_O increase of pressure.

#### Radiology

Anteroposterior CXRs before and within 24 hours after treatment with rhDNase were coded, blinded and interpreted randomly by two independent pediatric radiologists. Since a validated scoring system for atelectasis is lacking, a scoring system based on available literature [15-17] and personal experience of our radiologists was defined as follows.

Each X-ray was scored for atelectasis, hyperinflation and mediastinal shift. The presence or absence of hyperinflation was marked as 1 point or 0 points, respectively. The presence or absence of a mediastinal shift was scored as 1 or 0. Atelectasis was scored for each lobe. A partial atelectasis of one pulmonary lobe was scored as 1 point, and complete atelectasis of one lobe was marked as 2 points. The distinction between infiltrate and atelectasis was left up to the pediatric radiologist, and was judged similarly to that in routine clinical care. These results were summed for each CXR. The CXR score before rhDNase treatment was compared with the CXR score within 24 hours after treatment.

## Results

### Treatment

Conventional treatment of atelectasis before the use of rhDNase consisted of nebulised bronchodilators in 25 patients, nebulised NaCl 0.9% in 16 patients, systemic or inhaled glucocorticoids in 18 patients and physiotherapy in all patients.

RhDNase was nebulised in 18 patients and was given as a droplet in 12 patients on mechanical ventilation. All patients received antibiotics after obtaining the appropriate cultures because bacterial infections could not be ruled out and these children had elevated inflammatory markers and were seriously ill.

### RhDNase administration

**Figure 1:**
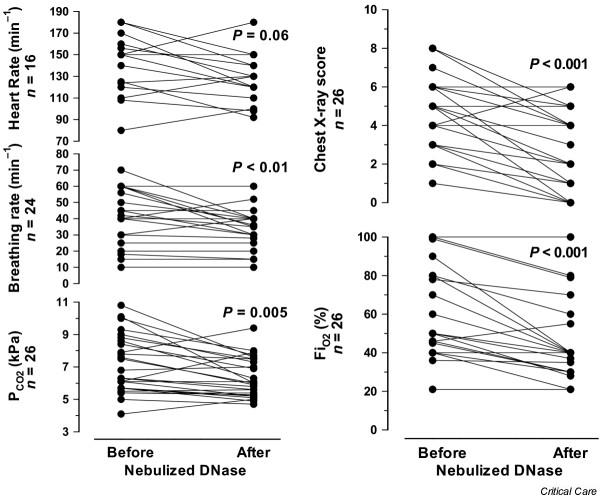
Change of cardiorespiratory parameters before and after treatment. Individual changes of the heart rate, breathing frequency, blood pCO_2 _and FiO_2 _before and within 2 hours, and the chest X-ray score before and within 24 hours after administration of DNase in 30 children treated for atelectasis. Although changes were statistically significant, a significant overlap was present before and after recombinant human DNase treatment (see also Table 1).

In 25 out of 30 patients who met the inclusion criteria, paired data of at least three cardiorespiratory endpoints were available. Individual values before and after rhDNase treatment are shown in Fig. 1. Group results are presented in Table 2. All clinical variables, except the HR, show a significant improvement within 2 hours following rhDNase treatment (*P *< 0.01, Fig. 1).

Anteroposterior CXRs before and within 24 hours after treatment were obtained in 22 of 30 patients. On average, the median CXR score improved from 4.0 to 2.0 (*P *< 0.001). No paired CXRs were available in eight patients. In seven of these eight patients, the hospital records documented that no CXR was made after rhDNase treatment because the clinical improvement was very obvious and another CXR was unnecessary. One patient died before a post-treatment CXR was made when all treatment, including mechanical ventilation, was discontinued because of the very poor prognosis of her mitochondrial encephalomyopathy. Three patients showed complete resolution of all atelectasis within 24 hours. Individual CXR improvement was observed in 17 patients, no clear change was seen in two patients and deterioration was observed in three patients. Agreement between the CXR scores by the two observers expressed as Cohen's kappa was 0.61 (*P *< 0.001).

Individual improvement of at least two endpoints was seen in 17 patients, but no clinical change was observed in five patients. Three patients on mechanical ventilation (two ex-premature infants now 6 months and 7 months corrected postgestational age with RSV bronchiolitis, and one 6-month-old full-term child with congenital airway narrowing with an adenovirus respiratory infection) showed an immediate deterioration after administration of rhDNase. This was possibly due to excessive mobilisation of mucus leading to temporary increased airway obstruction. Oxygenation was extremely difficult for 2 hours after administration of rhDNase in two of these patients. Oxygen saturations remained between 85% and 90%, despite increased ventilator settings. No other side effects were observed.

Twelve of the 16 patients on mechanical ventilation were extubated within 6 days after the onset of rhDNase treatment; six of these were extubated the day after administration of rhDNase. Four patients remained on mechanical ventilation for longer than 10 days.

## Discussion

This retrospective series, being the largest study so far, suggests that rhDNase may be an effective drug in the treatment of refractory atelectasis in non-CF patients. Quick resolution of atelectasis is important to reduce the number of days on mechanical ventilation with associated morbidity and to prevent the need for therapeutic bronchoscopy. Several case reports [10-14] and one randomised study on RSV bronchiolitis [5] suggest a clinical and radiological improvement after treatment with rhDNase in patients with atelectasis. In the present study rhDNase was administered when an infection was proven or suspected, and when patients had severe respiratory problems due to atelectasis.

Individual improvement of at least two endpoints was observed in 17 patients, and mobilisation of sputum occurred so rapidly in three children that this resulted in temporary worsening of the ventilation perfusion mismatch due to increased airway obstruction. Clinical improvement was observed in most of the children within 2 hours, and the mean values of the RR, pCO2 and FiO_2 _all improved significantly after rhDNase treatment. One could argue that the improvement of these respiratory parameters is statistically significant, but is not clinically relevant. However, the extubation rate and CXRs do suggest a clinically relevant effect. A substantial number of ventilated patients (six out of 16) with refractory atelectasis could be extubated within 24 hours following the first dose of rhDNase, and CXRs improved in 17 out of 22 cases within 24 hours. As in the study by Nasr and colleagues [5], CXRs were scored by two independent radiologists, thereby preventing observer bias. Agreement between the two pediatric radiologists can be considered satisfactory. Furthermore, the degrees of improvement of CXRs scored by each radiologist were also similar. Nasr and colleagues studied CXRs in infants with RSV bronchiolitis before and after treatment with rhDNase [5]. It is difficult to compare their results with those of the present study as they interpreted the change in CXR on admission and at discharge, they administered rhDNase only once daily and they used a different CXR score, reflecting CXR features of bronchiolitis. They found a small but significant improvement of the CXR score after rhDNase, while their control group showed a significant deterioration.

The deterioration with increased airway obstruction and ventilation-perfusion mismatch in three children on mechanical ventilation was interpreted as a result of rapid mobilisation of mucus in these three patients. This deterioration was observed in three out of 12 infants, but not in the seven children who were younger than these three patients. In a subanalysis no relationship was found between this deterioration and viral infection. We speculate that this deterioration may be due to the mode of administration. If the drug is instilled, the effective lung dose may be far greater than when it is inhaled; in all three patients, rhDNase was instilled endotracheally. Instillation may be an attractive option in that nebulised rhDNase administration in patients on mechanical ventilation results in significant deposition of the drug in the ventilator tubing, but it may also imply a risk. To prevent deterioration following instillation, however, lower starting doses of instilled rhDNase may be warranted. The dose we used for instillation was higher than reported by Boeuf and colleagues [11] and was lower than that administered bronchoscopically by Durward and colleagues [12]. Incidentally, in the case reports mentioned earlier, no clinical deterioration was observed when rhDNase was nebulised or was instilled bronchoscopically. In the randomised clinical trial on RSV bronchiolitis [5], a beneficial effect and no adverse events were observed. This was possibly also explained by using nebulised rhDNase rather than instilled rhDNase. In the present study, none of the known adverse effects such as pharyngitis, airway irritation, laryngitis, conjunctivitis or rash were observed, nor any rebound effects within 24 hours following administration.

There are several limitations to this study. First, it is a retrospective and open study potentially suffering from selection effects, and lacking a control group. Second, there are no validated scoring systems for atelectasis on CXR. Third, the sputum DNA content was not known, and rhDNase was administered irrespectively. Hence, the present study does not provide ultimate proof that improvement should be attributed to rhDNase treatment.

However, an association between drug treatment and a beneficial clinical response is more likely to be causal when the response follows immediately or quickly after administration of the drug, when the response is consistent, when the response is marked and when the response is plausible with respect to the pathophysiology behind the disorder. In addition, the quick alterations following rhDNase administration are also consistent with *in vitro *experiments that demonstrated a quick effect on sputum characteristics [1,18]. We therefore think that there is a causal relationship between DNase administration and the clinical outcome, but further randomised control trials are required to confirm this.

In addition, because rhDNase is expensive, a cost-benefit calculation in such a trial is warranted. Effective treatment of atelectasis is likely to reduce the stay in the hospital. Daily treatment costs of €60 should ideally reduce the length of hospital stay and outweigh the costs of hospital stay (which would be €1000/day in The Netherlands).

## Conclusion

We conclude that our observations suggest efficacy of the drug in at least 17/25 (68%) of the patients, and show complete resolution of all atelectasis in three patients within 1 day. RhDNase may hence have a place in the treatment of children with atelectasis. However, randomised controlled studies are needed to prove this, and also to assess whether it is cost-beneficial and can shorten the hospital stay of children with atelectasis.

## Key messages

• 	Rapid clinical and radiological improvement was observed in the large majority of children following rhDNase treatment for atelectasis.

• 	Increased airway obstruction and ventilation-perfusion mismatch may occur when rhDNase is instilled endotracheally, possibly due to rapid mobilisation of mucus.

• 	DNase may be an effective and cost-beneficial treatment for atelectasis in non-CF pediatric patients.

## Abbreviations

CF = cystic fibrosis; CXR = chest X-ray; FiO_2 _= Fraction of inspired Oxygen ; HR = heart rate; pCO_2 _= Pressure of CO_2_; rhDNase = recombinant human DNase; RR = respiratory rate; RSV = respiratory syncytial virus

## Competing interests

The author(s) declare that they have no competing interests.

## Authors' contributions

TH performed data analysis and contributed to the manuscript. MdH provided clinical care and contributed to the manuscript. MHL performed radiology data analyses and designed the study. ASD performed radiology data analyses and designed the study. PJFM performed data analysis and contributed to the manuscript.

## Figures and Tables

**Table 1:** Demographic data of the study group

Sex (male/female)	19/11
Median (range) age	1.6 years (14 days–12 years)
Median (range) duration of disease before atelectasis	12 days (2–365 days)
On ventilator/no ventilator	16/14
Intensive care/medium care	25/5

**Table 2:** Clinical variables before and after administration of recombinant human DNase

Variable	Before	After	*P*
Heart rate (beats/min)	150 (121–164)	130 (115–145)	0.06
Respiratory rate	41 (26–60)	36 (25–40)	0.01
PCO_2 _(kPa)	6.55 (5.7–8.6)	6.00 (5.3–7.5)	0.005
FiO_2 _(%)	50 (40–100)	40 (21–75)	<0.001
Chest X-ray score	4 (2–6)	2 (0–4)	<0.001

Data presented as the median (interquartile range). Comparisons were made using the Wilcoxon signed rank test.
